# Probability of lek collapse is lower inside sage-grouse Core Areas: Effectiveness of conservation policy for a landscape species

**DOI:** 10.1371/journal.pone.0185885

**Published:** 2017-11-09

**Authors:** Emma Suzuki Spence, Jeffrey L. Beck, Andrew J. Gregory

**Affiliations:** 1 School of Earth Environment and Society, Bowling Green State University, Bowling Green, Ohio, United States of America; 2 Department of Ecosystem Science and Management, University of Wyoming, Laramie, Wyoming, United States of America; Oregon State University, UNITED STATES

## Abstract

Greater sage-grouse (*Centrocercus urophasianus*) occupy sagebrush (*Artemisia* spp.) habitats in 11 western states and 2 Canadian provinces. In September 2015, the U.S. Fish and Wildlife Service announced the listing status for sage-grouse had changed from *warranted but precluded* to *not warranted*. The primary reason cited for this change of status was that the enactment of new regulatory mechanisms was sufficient to protect sage-grouse populations. One such plan is the 2008, Wyoming Sage Grouse Executive Order (SGEO), enacted by Governor Freudenthal. The SGEO identifies “Core Areas” that are to be protected by keeping them relatively free from further energy development and limiting other forms of anthropogenic disturbances near active sage-grouse leks. Using the Wyoming Game and Fish Department’s sage-grouse lek count database and the Wyoming Oil and Gas Conservation Commission database of oil and gas well locations, we investigated the effectiveness of Wyoming’s Core Areas, specifically: 1) how well Core Areas encompass the distribution of sage-grouse in Wyoming, 2) whether Core Area leks have a reduced probability of lek collapse, and 3) what, if any, edge effects intensification of oil and gas development adjacent to Core Areas may be having on Core Area populations.

Core Areas contained 77% of male sage-grouse attending leks and 64% of active leks. Using Bayesian binomial probability analysis, we found an average 10.9% probability of lek collapse in Core Areas and an average 20.4% probability of lek collapse outside Core Areas. Using linear regression, we found development density outside Core Areas was related to the probability of lek collapse inside Core Areas. Specifically, probability of collapse among leks >4.83 km from inside Core Area boundaries was significantly related to well density within 1.61 km (1-mi) and 4.83 km (3-mi) outside of Core Area boundaries. Collectively, these data suggest that the Wyoming Core Area Strategy has benefited sage-grouse and sage-grouse habitat conservation; however, additional guidelines limiting development densities adjacent to Core Areas may be necessary to effectively protect Core Area populations.

## Introduction

Greater sage-grouse (*Centrocercus urophasianus*; hereafter ‘sage-grouse’) are a landscape species of conservation concern across 11 U.S. states and 2 Canadian provinces [1, 2, 3]. Concern about declining populations of this iconic sagebrush (*Artemisia* spp.) obligate species was first noted 100 years ago [4] and has been brought to the forefront of conservation efforts in western states and provinces over the past three decades [3, 5]. Sage-grouse were nominated for listing under provisions of the Endangered Species Act (ESA) eight times from 1999–2015. In 2010, during the seventh listing attempt, sage-grouse were deemed to be *warranted but precluded* [6]. This decision was based on several factors, including habitat loss, fragmentation and inadequacy of existing regulatory mechanisms [6]. Following this attempt, the 11 states within the current range of sage-grouse took steps to either fully implement previously established sage-grouse management plans or to develop and implement new plans [7]. The eighth, and most recent, ESA listing decision in 2015 resulted in a decision of *not warranted*, in part because of new regulatory mechanisms implemented to protect sage-grouse [7].

The 2015 ESA finding called out three state plans—Wyoming, Oregon and Montana—to be specifically highlighted because “they provide the greatest degree of regulatory certainty in addressing potential threats on State and private lands [7].” Of these three states, Wyoming represents 64% of the extant sage-grouse population in their eastern range [8] and is centrally located within the U.S. distribution of sage-grouse [9]. Range-wide, Wyoming encompasses about 37% of the male sage-grouse population, 25% of habitat, and more leks than any other state [8]. As such, conservation of sage-grouse in Wyoming is critical to the species.

The most prevalent threat to sage-grouse in Wyoming is loss and degradation of habitat due to energy development, especially from oil and gas extraction [10]. Numerous studies have documented localized and regional declines in sage-grouse populations attributable to activities directly associated with oil and gas extraction [3, 11, 12]. More specifically, high intensity drilling (≥3.1 wells/km^2^) in a previously undeveloped area resulted in a 61% reduction in the total number of males counted in northeast Wyoming [13]. In addition, probability of lek persistence fell below 50% in north-central Wyoming when oil and gas well densities exceeded >2 wells/km^2^ within 1.0 km around leks [14]. It has also been found that, leks that had ≥1 oil or gas well within a 0.4-km (0.25-mile) radius encircling the lek had 35–91% fewer attending males than leks with no well within this radius [15]. While precise mechanisms underlying population decline in areas with active oil and gas development are still under investigation, it has been demonstrated that: yearling males avoid recruiting to leks near oil and gas infrastructure; yearling females avoid nesting within 950 m of infrastructure; both yearling males and females have lower annual survival near infrastructure; and yearling males are less likely to establish breeding territories in areas with infrastructure [16]. As such, effective sage-grouse conservation efforts in Wyoming must consider the impacts of oil and gas development on sage-grouse populations.

Energy production is a major industry in Wyoming. The petroleum industry has been exploring oil and gas resources in Wyoming for over 130 years, with 22 of Wyoming’s 23 counties producing combinations of coal, crude oil, or natural gas [17]. The oil and gas industry also is a leading employer in Wyoming, peaking in 1981 at a total employment of 32,000 and employing 25,800 directly in 2014 [17]. Furthermore, oil and gas activities in Wyoming generate substantial economic output that has direct impact on the United States economy. It has been estimated that oil and gas activities contributed $18.6 billion in economic output, equaling 32% of the state’s total economic output or gross revenues in 2007 alone [18]. Consequently, listing sage-grouse or other species that may conflict with energy development under the ESA will incur economic impacts on the state.

**Fig 1:**
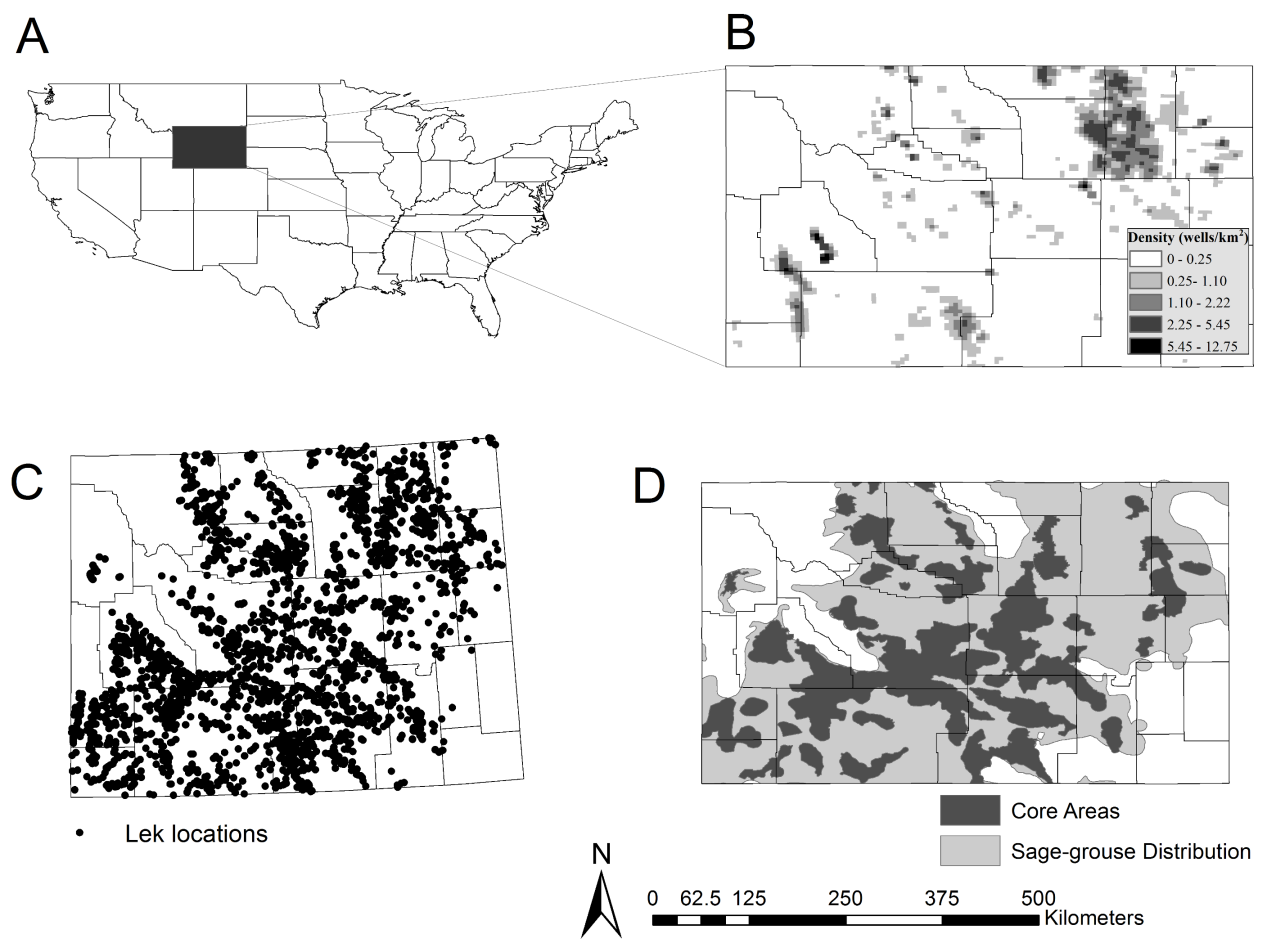
**(A) Wyoming in relation to the United States. (B) Density of average oil and gas wells per km**^**2**^ **(C) 31 sage-grouse Core Areas designated by The State of Wyoming SGEO (version 3) within the distribution of sage-grouse (D) Locations of 2,382 leks archived within the lek database.** *For our* study, only leks surveyed from 1999 to 2013 were used. Collectively, these were the data sets that were used to conduct our analysis.

In 2008, with the seventh ESA listing attempt underway, then Wyoming governor Freudenthal, enacted the Sage-Grouse Executive Order (SGEO) [18]. The Core Area Strategy established by the SGEO set aside 31 distinct Core Areas encompassing 24% of Wyoming (Fig 1C), to contain a goal of 67% of the state’s breeding population of male sage-grouse [19, 20]. Under direction of the Wyoming SGEO, Core Areas are managed with limitations on future anthropogenic disturbance including seasonal timing stipulations and limiting surface disturbance to an average of 5% or 1 well per 2.6 km^2^ (1 mi^2^) within suitable habitat in project impact analysis areas [19, 20]. However, Gamo and Beck [21] reported surface disturbance in impact analysis areas averaged 6.4% (range = 0.7–18.7%) within 19 of 31 (61.3%) Core Areas across the state where surface disturbance was quantified from 2012–2014.

Practices embodied within the SGEO are now receiving greater assessment for their effectiveness in conserving sage-grouse and their habitats (see [21, 22, 23]). Since establishment, the SGEO and Core Areas have been re-authorized and revised three times [8]. At its inception, Core Areas were delineated to encompass most of the breeding population of sage-grouse in Wyoming; however, the efficacy of Core Areas in maintaining statewide breeding populations of grouse as designed has only recently been assessed since Core Areas were implemented in 2008 [21]. Other studies have evaluated the efficacy of Core-Areas for sage-grouse conservation by sampling at smaller spatial and temporal scales to address specific life-history components influencing sage-grouse movements, demography, and habitat selection [21, 23, 24]. Moreover, oil and gas wells increased in Wyoming nearly four-fold from 1991 to 2011 [12], and wells in non-Core Areas increased 29 times more than in Core Areas throughout the statewide sage-grouse range from 1986 to 2014 [21]. Prior to our study, no studies had evaluated lek collapse in Core and non-Core Areas nor the degree to which intensive land use adjacent to Core Areas may be impacting sage-grouse population viability within Core Areas. Thus, we sought to directly assess: 1) the extent that Core Areas encompass the distribution of sage-grouse in Wyoming, 2) whether Core Area leks have a reduced probability of lek collapse (a new metric that was created for this study) compared to non-Core Area leks, and 3) what, if any, edge effects the intensification of oil and gas development adjacent to Core Areas may have had on Core Area sage-grouse populations.

## Methods

### Study system

Our analysis took place at the spatial extent of the distribution of sage-grouse in Wyoming (Fig 1A), which matched the management scale for the SGEO (Fig 1C). Wyoming has a mean elevation of 2,042 m, but is topographically complex with majestic mountains and high plains. The variation of elevation across the state makes it difficult to divide it into homogeneous climatic regions, while its latitude predisposes the state to a relatively cool climate. Annual precipitation across Wyoming is highly variable due to its widely varying topography. The dry cool climate in lower elevation areas with nutrient-poor soils limit natural vegetation in rangeland communities to hardy plants including sagebrush (*Artemisia* spp.), black greasewood (*Sarcobatus vermiculatus*), rabbitbrush (*Chrysothamnus* and *Ericameria* spp.), saltbush (*Atriplex* spp.), sod-forming grasses, and bunchgrasses [25].

### Data

We used the Wyoming Game and Fish Department’s (WGFD) sage-grouse lek database to characterize sage-grouse populations across Wyoming (all data request inquiries should be directed to WGFD research staff at https://wgfd.wyo.gov). The WGFD has conducted annual counts of male sage-grouse attending individual leks or lek complexes since 1948. Sage-grouse are a gallinaceous bird with a gregarious lek-mating system [26]. Leks are typically located at a nexus of needed resources and are spatially correlated with nest sites; 64% of nests occur within 5 km of leks and 92.5% of nests within 8.5 km of leks in relatively contiguous sagebrush habitats in central and southwest Wyoming [27]. Trend monitoring and research studies often use average males per lek as a relative index of sage-grouse populations [15, 28, 29, 30]. While research suggests year-to-year variation in lek counts is high or that lek count data have high periodicity [30], lek counts have been shown to be an accurate account of long-term population trends, particularly over a broad geographic extent [29, 30]. Trends in male sage-grouse counts have also been shown to be a reasonable index of population change [31, 32]. Therefore, the WGFD’s annual lek count data is suitable for use in our analysis of long-term population trends and broad-scale population response to land use and management.

Following WGFD protocols, lek counts occurred between one half hour before and two hours after sunrise from 15 March to 30 April from 1999 to 2013. Surveyors reported numbers of male sage-grouse attending leks from multiple counts during each survey, with each lek surveyed at least once and up to three times per breeding season, with the highest surveyed number being reported. We used the maximum count from multiple counts per year and confined our analysis to the years 1999 to 2013 because this represents a balanced study design, meaning similar time included pre- (2001–2007, 7 years) and post- (2008–2013, 6 years) SGEO establishment. Furthermore, data from this time are more robust than earlier years in the dataset, and 2013 represents the latest year that data were available during this study. The total number of leks included in our analysis was 2,382 (Fig 1C).

To characterize oil and gas well development across the state, we used Wyoming Oil and Gas Conservation Commission (WOGCC; all data request inquiries can be made at: www.wogcc.wyo.gov) data from 1948 to 2013. The WOGCC is a public database that provides locations of every oil and gas well of any status across Wyoming with the date that a drilling rig was activated, as well as the last date active and the date capped (if capped) [33]. For the purposes of our analysis, we included only those wells for which the WOGCC database indicated a physical structure was still present on the landscape; we did not differentiate between producing or abandoned wells. The reason for doing this was two-fold: the juxtaposition of producing and abandoned wells is such that producing wells and abandoned wells are intermixed on the landscape and the data used has lots of missing data values, where it was often unknown if the well was still producing. The abandoned wells likely have similar levels of human traffic because of neighboring producing wells and we did not want to add precision to the data set that we did not feel we had. The total number of wells included in the analysis was 72,562, which occurred at highest densities in northeast and portions of southwest Wyoming with densities reaching as high as 11.33 well/km^2^ (Fig 1D).

### Analysis

We performed all spatial analysis in ArcMap 10.3 [34] and all statistical analysis in R [35]. Summary statistics were calculated to assess lek attendance in and out of Core Areas, across Wyoming for each year from 1999 to 2013. Total leks, total birds, birds/lek, minimum birds, maximum birds, number of leks with zero counts, and the percentage of total leks with zero counts were calculated for each year independently for all leks inside and outside Core Areas. Unless otherwise stated, we present all lek counting data ±1 standard error.

Lek count data were also used to create an ancillary data set that evaluated the probability of lek collapse over time. For the purpose of our analysis, we defined lek collapse as a lek where zero birds were observed at the lek site for three consecutive years; i.e., if there were more than zero birds counted on a particular lek in any of the three years in question, that lek was not collapsed. Data were compiled on the basis of the year in question and the two previous year’s data. Only leks that had data for all three years were included in the analysis, allowing for a variable number of leks included in each year. Due to this data structure, the first year accessed for lek collapse was 2001, and the last year was 2013. Additionally, each year of the study was analyzed independently from all other years; therefore, all recoveries were independent from collapse. We used a Bayesian conditional binomial probability analysis to estimate the conditional probability of lek collapse conditioned on a lek being a Core or non-Core lek using package binom using uninformed priors [36]. The probability was determined using the total number of leks that were collapsed in a given year and the total number of leks observed on the landscape in that same year. A Bayesian approach was used because it handles large data sets better than frequentist approaches.

Lastly, we evaluated whether there was a boundary effect attributable to oil and gas well development immediately outside Core Area boundaries affecting Core Area populations’ probabilities of lek collapse *inside* Core Areas. To test this, we ran pairwise linear regressions of the probability of lek collapse (across all years in the study) versus oil and gas well densities (Fig 1B) within different buffered regions *outside* of the Core Area boundary. The average probabilities of collapse that were used were: all leks inside the core (core-leks); only those core-leks located within 1.61 km (1 mi) of the boundary; leks located inside the Core Area within 4.83 km (3 mi) of the boundary; and what we term “Core-Core” leks, which were those leks located >4.83 km away from the Core Area boundary. The use of U.S. statute miles were used to make the application of any relevant findings easier, as miles are what are used by most wildlife management agencies. We measured oil and gas well densities within 1.61-km and 4.83-km buffered regions *outside* the Core Area boundary. We used oil and gas well density from the year prior to the lek count year, because that reflected the completed wells on the landscape that could have affected the population of sage-grouse in the year they were counted. We calculated the density (number of wells per km^2^) of oil and gas wells using Focal Statistics in ArcInfo 10.3 and ran linear regressions using package regress [37] in R [35].We then applied the linear model used in the regression analysis to predict percent change in lek attendance within Core Areas as a function of development density adjacent to the core area.

## Results

**Fig 2:**
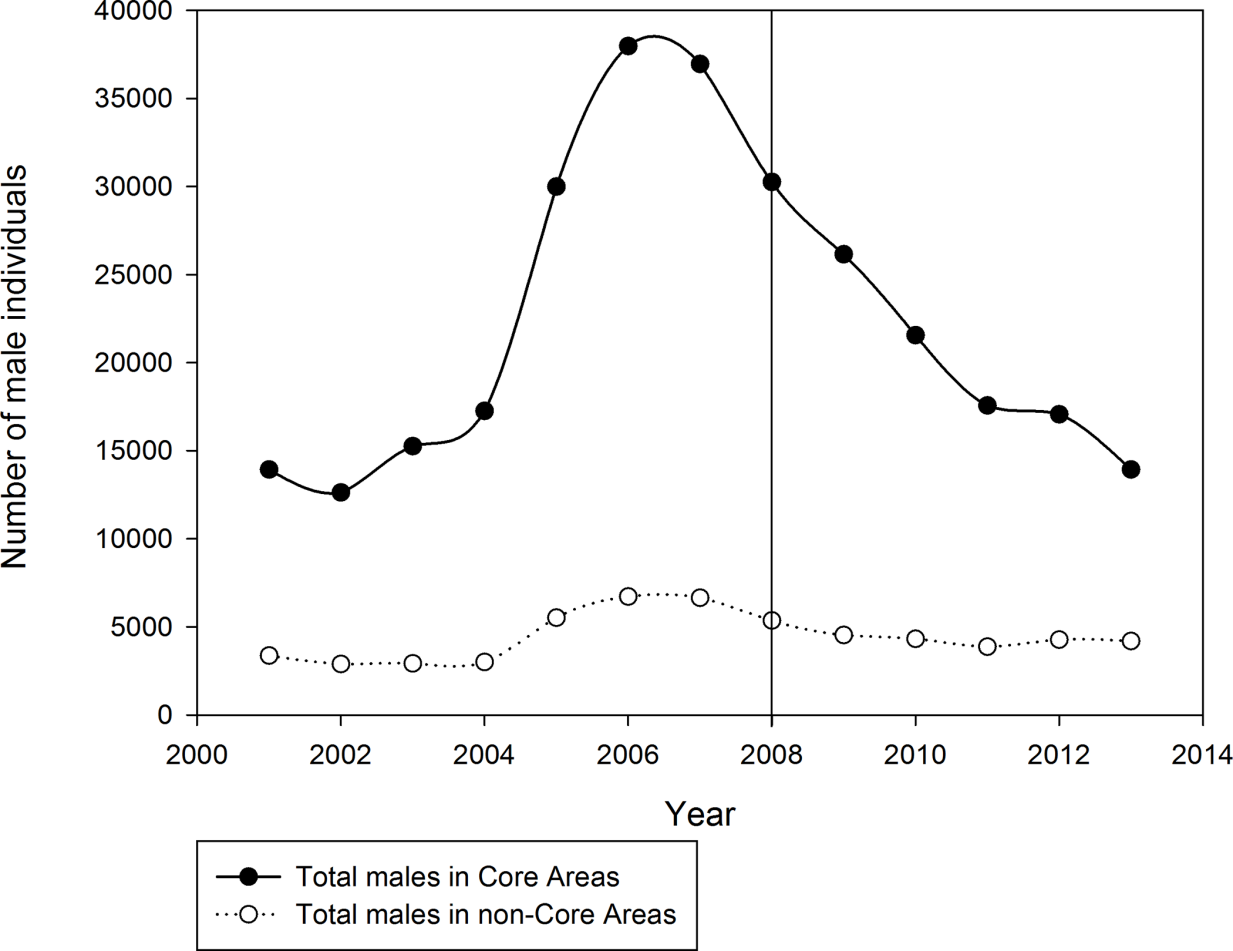
Observed population trends from male lek counts inside and outside of Core Areas, by year, from 2001–2013 in Wyoming. The vertical line at 2008 indicates the year in which the SGEO was enacted.

**Fig 3:**
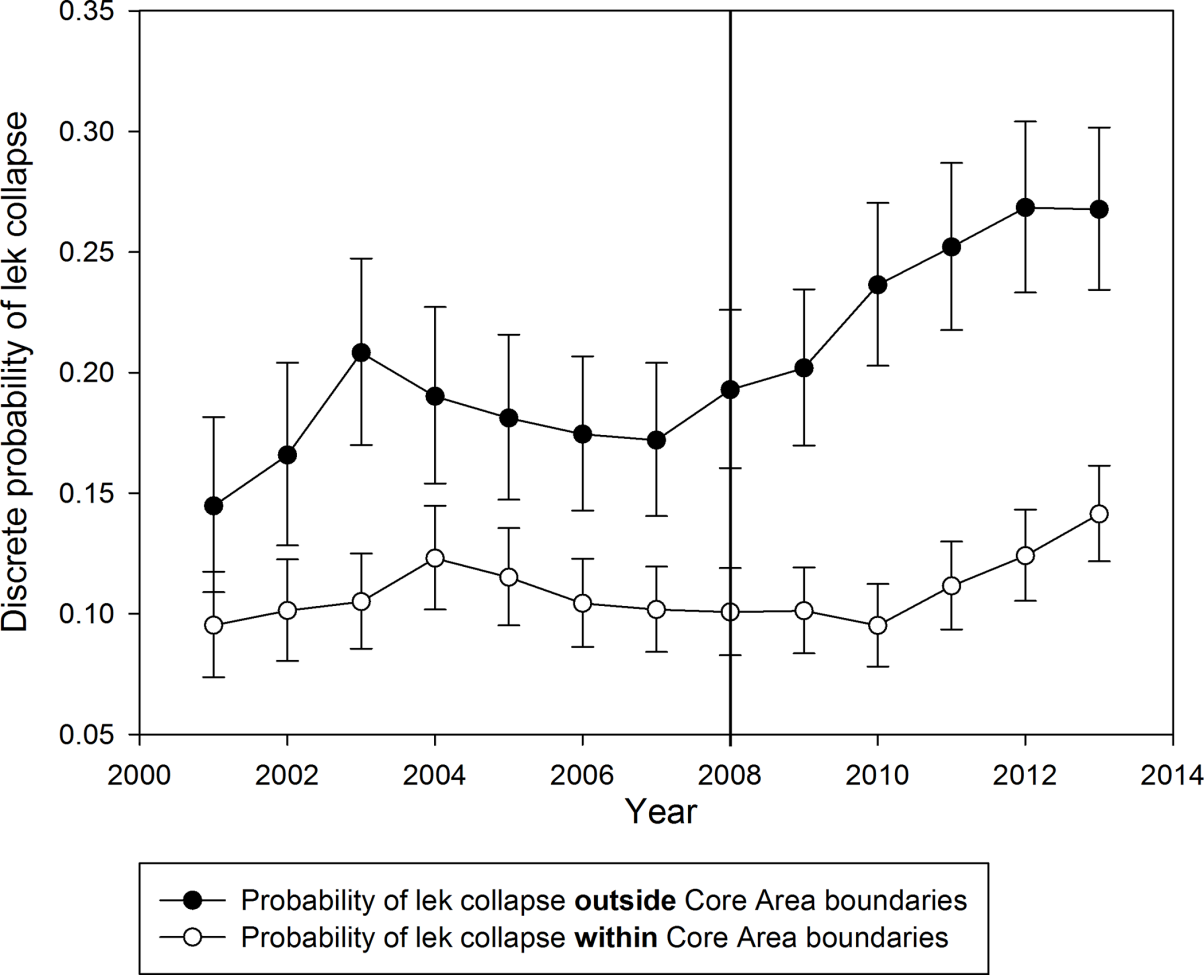
Probability of lek collapse was calculated independently for each year using a Bayesian binomial probability function of the Core Area leks and non-core area leks in Wyoming, 2001–2013. All probabilities are independent probabilities of collapse, however probabilities have been displayed as continuous for easier viewing. The vertical line at 2008 indicates the year in which the SGEO was enacted. The 95% credibility interval is given for each year.

Wyoming Core Areas included ~66% of active leks and accounted for ~83% of male sage-grouse attending leks in Wyoming from 1999 to 2013 (Table 1) (Fig 2). Across all years, an average of 82% of males in Wyoming were located in Core Areas (range = 76–85%; Fig 2). In addition, there was higher male attendance on Core Area leks than non-Core Area leks (average males/core lek = 22 ± 2; average males/non-core lek = 9 ± 1; two sample t_2,363.8_ = -14.75, P <0.001; Table 1). The probability of lek collapse was higher in non-Core Areas (average = 20.7%) than in Core Areas (average = 10.9%; Fig 3). After 2001, the credibility intervals surrounding the probability of lek collapse did not overlap, save 2001, further suggesting that the likelihood of lek collapse was greater outside of Core Areas than inside (Fig 3).

**Table 1:** Summary statistics of male lek counts for greater sage-grouse in Wyoming, USA, 1999–2013.

Leks	Average total leks	Range in total leks evaluated	Average percent of total leks	Average males/lek	Average percent of total males	Average total leks with 0 males	Average percent of total leks with 0 males
**Core**	**1,013±43**	**688–1177**	**66±0.37**	**22±2**	**83±1**	**300±16**	**30±1**
**Non-Core**	**518±27**	**355–662**	**33±0.37**	**9±1**	**17±1**	**254±17**	**49±1**
**Total**	**1530±69**	**1043–1939**	**1±0**	**18±2**	**1±0**	**534±31**	**36±1**

Averages are reported to nearest whole number ± 1 SE.

**Fig 4:**
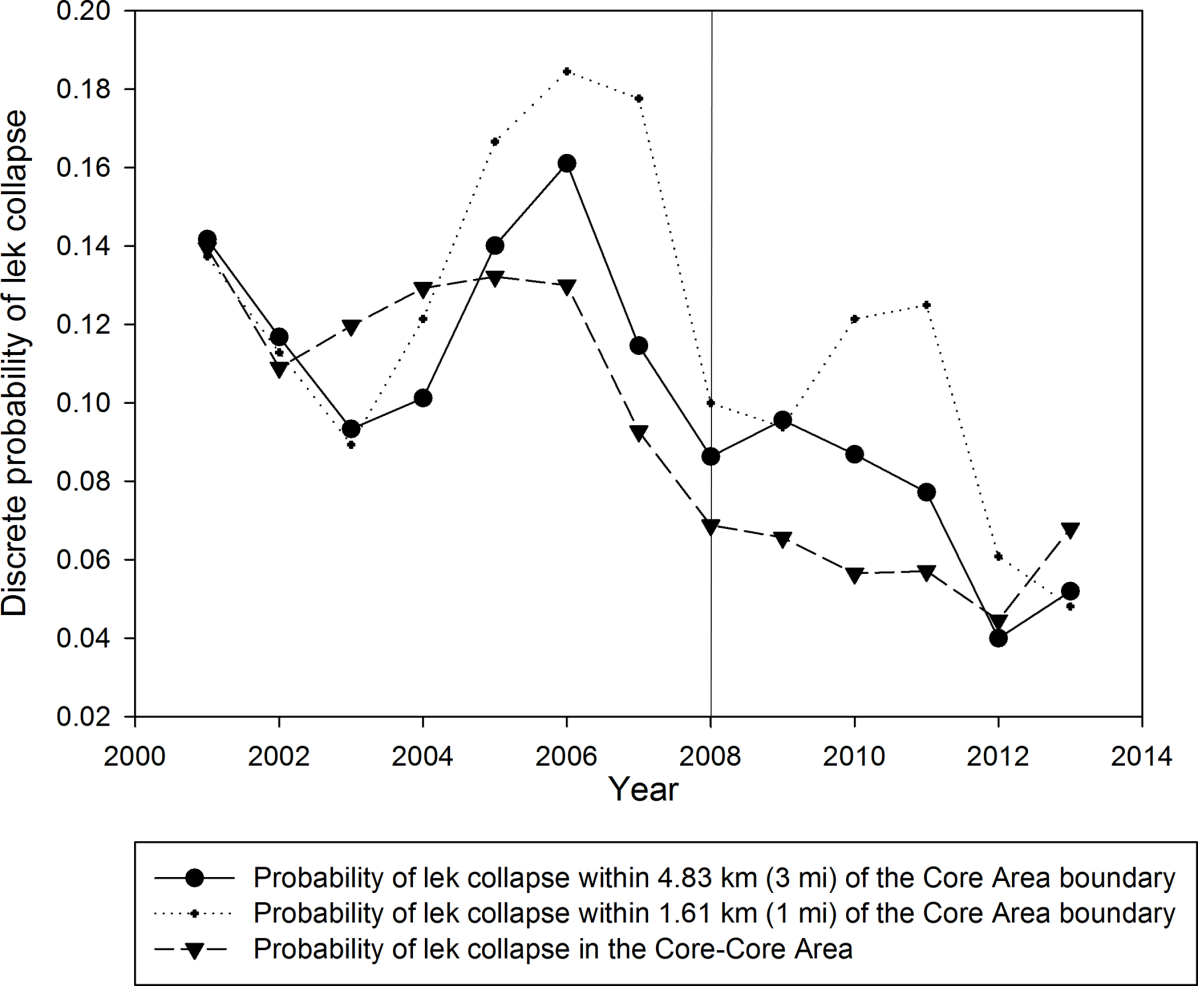
Probability of lek collapse was calculated using Bayesian binomial probability from 2001–2013 in Wyoming based on leks located 1.61 km (1 mi), 4.83 km (3 mi) outside Core Areas and the Core-Core Area. All probabilities are independent probabilities of collapse; however probabilities have been displayed as continuous for easier viewing. The vertical line at 2008 indicates the year in which the SGEO was enacted. The 95% credibility intervals have not been included, but all CI’s overlap.

**Fig 5:**
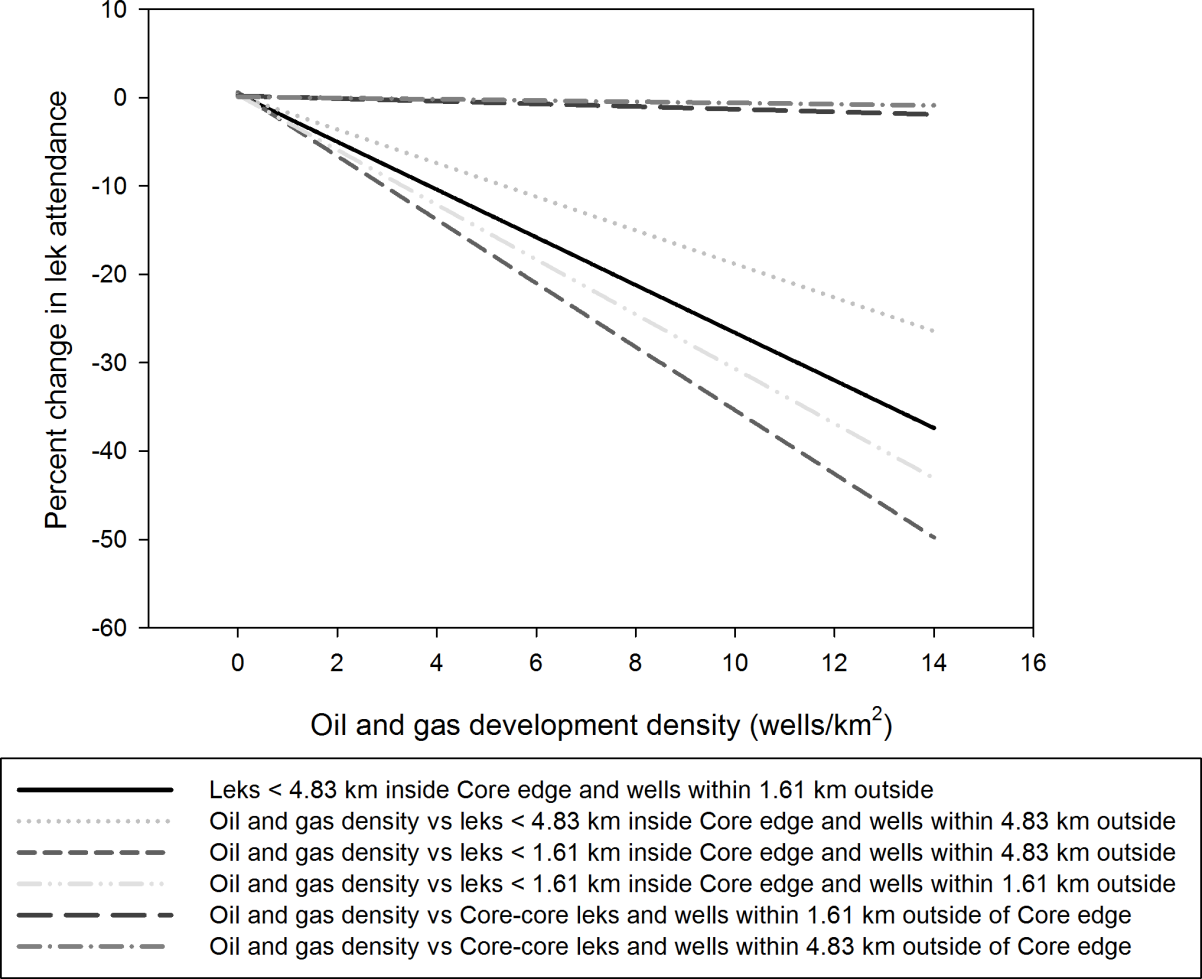
Predicted percent change in Core Area lek attendance as a function of oil and gas development adjacent to Core Area boundaries. We used the linear model from the regression of Core Area lek attendance for leks within 1.61 km and 4.83 km of the Core Area boundary against oil and gas development density within 1.61 km and 4.83 km outside of the Core Area boundary to predict the percent decline in lek attendance of Core Area leks.

Plots of the probability of lek collapse, by year, for leks in Core Areas <1.61 km from Core boundaries and for Core-Core Areas (i.e., leks >4.83 km from Core Area boundaries) showed 2–4 year periodicity (Fig 4). From 2001–2013, Core-Core Area leks had a lower average probability of lek collapse (9.2%) than leks <1.61 km from Core Area boundaries (11.8%), but the credibility intervals overlapped each year indicating no difference (Fig 4). Across the observed range of oil and gas well development densities adjacent to Core Areas we observed differences in the predicted change in lek attendance from 0 to -54% (Fig 5). The steepest rate of decline was associated with leks adjacent to the Core Area boundary and oil and gas well density within 4.83 km outside of the boundary. The least steep rate of decline was associated with leks located ≥4.83 km inside the Core Area boundary and oil and gas well density within 1.61 km outside the Core Area boundary. Collectively these results suggest that development density adjacent to the Core Area negatively impacted lek attendance inside the Core Area (Fig 5).

Oil and gas well densities inside Core Areas were not related to the probability of lek collapse in Core Areas (r^2^ = 0.02, P = 0.67). However, development density outside Core Area was related to probability of lek collapse inside Core Area (Table 2). We regressed the probability of lek collapse of the Core-Core leks against oil and gas well development density outside the Core Area boundary at 1.61 km and 4.83 km and found a strong, negative trend (P<0.00, β_4.83 km_ = -0.714; β_1.61 km_ = -1.009; Table 2). Furthermore, when the probability of collapse for leks located inside Core Area within 4.83 km of the boundary was regressed against the same development densities as Core-Core leks (4.83 km and 1.61 km), these were also significant and negative (P_4.83 km_ = 0.011, β_4.83 km_ = -0.504; P_1.61 km_ = 0.013, β_1.61 km_ = -0.707; Table 2). Using the logit link function to predict the change in Core Area lek attendance as a result of development densities within 1.61–4.83 km of Core Area boundaries indicated declines of 0–36% decline across the range of observed oil and gas well development density (Fig 5). In general, Core Area lek declines within 1.61 and 4.83 km of the Core Area boundary were modest 0–10% (0–2 birds/lek) until oil and gas well density adjacent to Core Area boundaries exceeded 2 well /km^2^ (Fig 5).

**Table 2:** Linear regression results of probability of lek collapse within Core Areas versus oil and gas well development densities (DD) outside of Core Area in Wyoming, USA, 2001–2013.

Model	β	SE	r^2^	F-value	P
**All Core leks vs. 4.83 km DD**	**0.035**	**0.067**	**0.027**	**0.278**	**0.609**
**All Core leks vs. 1.61 km DD**	**0.057**	**0.095**	**0.035**	**0.357**	**0.563**
**Core-Core leks vs. 4.83 km DD**	**-0.714**	**0.088**	**0.869**	**66.43**	<0.000*
**Core-Core leks vs. 1.61 km DD**	**-1.009**	**0.136**	**0.846**	**54.89**	<0.000*
**1.61 km Core leks vs. 4.83 km DD**	**-0.288**	**0.241**	**0.125**	**1.426**	**0.260**
**1.61 km Core leks vs. 1.61 km DD**	**-0.383**	**0.348**	**0.108**	**1.211**	**0.297**
**4.83 km Core leks vs. 4.83 km DD**	**-0.504**	**0.161**	**0.494**	**9.776**	0.011*
**4.83 km Core leks vs. 1.61 km DD**	**-0.707**	**0.235**	**0.375**	**9.047**	0.013*

The average probability of lek collapse used in this analysis was from leks located: in the entirety of the Core Area (All Core leks), within 1.61 km inside the Core Area boundary (1.61 km Core leks), within 4.83 km inside the Core Area Boundary (4.83 km Core leks) and greater than 4.83 km inside the Core Area Boundary (Core-Core leks). The average oil and gas well development densities (wells/km^2^) used in this analysis were the average oil and gas well density at 1.61 km outside the Core Area boundary (1.61 km DD) and 4.83 km outside the Core Area Boundary (4.83 km DD).

*Significant effect.

## Discussion

Our analysis suggests that protecting Sage-Grouse habitats under the auspice of the Wyoming Sage-Grouse Core Area Strategy is effective in conserving sage-grouse leks in light of broad-scale habitat loss and fragmentation due to oil and gas development. That is to say, the strategy of designating high quality habitat and limiting surface disturbances was protecting vital habitat for the species. Core Areas contained more leks, the majority of the extant sage-grouse population in Wyoming, and had a lower probability of lek collapse than non-Core Area leks (Fig 2) (Tables 1 and 2). That is to say that designated Core Areas overlapped well with the distribution of sage-grouse in Wyoming and that the Core Area Strategy aids in reducing the probability of lek collapse when compared to non-Core Area leks. As expected, the probability of collapse inside Core Areas differed from non-Core Area leks the entirety of our study, save 2001. This finding was predictable given how Core Areas were delineated to avoid existing energy disturbance and the low densities of disturbance where Core Areas were to be established prior to the SGEO in 2008. It is not surprising that the difference in the probability of collapse was different inside and outside Core Areas prior to the enactment of the SGEO because Wyoming’s Sage Grouse Implementation Team placed Core Areas in locations that historically had high quality habitat [21]. However, our results indicate that the difference in the probability of collapse increased more rapidly after the establishment of the SGEO. In light of increased oil and gas well development and the limitation of that development in Core Areas, this suggests that the SGEO is safeguarding critical sage-grouse habitats at the scale of the entire state of Wyoming.

One area of potential concern is that at first glance Core Area male lek attendance since 2008 was declining more rapidly than non-Core Area lek attendance (Fig 2). However, this is probably not a major conservation concern because, qualitatively speaking, the two trends (Core Area and non-Core Area) track each other, just the amplitude was much greater in Core Areas than outside of Core Areas (Fig 2). It is known that sage-grouse populations show a six-to-nine year periodicity [31]. With this in mind, it would appear as though sage-grouse populations residing in non-Core Areas had a dampened population oscillation. Blomberg and Coates [38, 39] have shown that sage-grouse respond strongly to high precipitation (boom years), and during dry years when leks collapse our data suggest that Core Area leks did not decline below levels observed in non-Core Area leks. Therefore, these observations suggest that Core Area populations were more resilient to environmental stochasticity than non-Core Area populations [40]. In comparison, Gamo and Beck [21] reported that consistently less variation in male lek counts in Core compared to non-Core Area leks in Wyoming suggested Core Areas leks were more stable and resilient to change. Moreover, since the enactment of the SGEO, Wyoming sage-grouse populations appear to have been in the decline stage of their population cycle [21]; however, sage-grouse populations in Wyoming and the West began increasing again in 2014 and 2015 [41].

We also detected that oil and gas development adjacent to Core Areas was negatively correlated with sage-grouse lek attendance inside Core Areas (Table 2). Specifically, the probability of lek collapse in Core Areas was positively associated with development density outside the Core Area. Similarly, the probability of lek collapse decreased as a function of distance from the Core Area edge. Areas within 1.61 km of the Core Area edge showed the lowest rate of decreased lek collapse, but were non-significant likely to due to small sample sizes. Similarly, when we evaluated the percent decline in lek attendance associated with oil and gas development outside of the Core Area, we saw the strongest response in percent decline in leks nearest the Core Area boundary and a non-significant decline in lek attendance for leks located ≥4.83 km from the core boundary (Fig 5). This suggests a possible edge effect of oil and gas development on sage-grouse lek attendance. Consequently, some Core Areas may be ineffective at protecting sage-grouse because they lack Core-Core areas, which is a common edge effect described in the ecological literature [42]. One possible explanation of this phenomena, is that high development density adjacent to Core boundaries may increase mortality for birds that venture outside of the Core Area or limit access to otherwise suitable habitat outside of Core Areas [43]. However, these areas could also be targets for mitigation and restoration work where restorative practices may be applied to mitigate impacts at other critical sites [44].

Some have criticized the SGEO, arguing that simply limiting anthropogenic disturbance associated with energy development does nothing to secure the high quality habitat necessary to protect the species. These critics argue that absent target sage-grouse habitat protection goals, the SGEO will not be effective [45, 46, 47]. In addition, these same entities also note the SGEO does not reduce existing oil and gas infrastructure on the landscape, but rather aims to slow the rate of oil and gas expansion [45, 46, 47]. Conversely, our data suggest that SGEO is providing broad protection to sage-grouse at the scale at which it was intended to function (statewide). Studies have shown relatively limited utility to SGEO when evaluating site-specific effects within a basin or oil field [22, 24]; however when accessing landscape scales, such as we have in this study, it may be essential to look at the importance of scale when evaluating landscape management plans such as the Wyoming SGEO [48, 49].

Dinkins et al. [23] concluded that habitat quality differences for female sage-grouse nesting in Core and non-Core Areas across multiple study areas in Wyoming most likely occurred at the landscape, rather than the microhabitat scale. Ecological processes interact in complex ways, rarely scale in space or time and thresholds are common [50, 51]. Recent studies with greater prairie-chickens (*Tympanuchus cupido*) have demonstrated that drivers of reproductive success and nest placement can vary in space and time [52], and that selected attributes at one location or time period might be avoided at another [53]. Similarly, Gregory and Beck [12] observed identical oil and gas development densities having differential impacts on sage-grouse lek attendance across a 10-year time period in Wyoming. This level of heterogeneity in response by grouse to human land use and land management suggests that traditional localized metrics of sage-grouse response to SGEO management may not scale state-wide and that attributes typically found to be important locally, may not make appropriate management targets for managing sage-grouse across a broad and ecologically complex landscape like Wyoming [54, 55].

Therefore, it is reasonable to infer that the Wyoming SGEO is effective in encompassing core habitat areas for sage-grouse and maintaining lek activity despite some criticism of its purpose or data suggesting that SGEO is not benefiting one particular demographic parameter at one localized population. SGEO was not intended to provide local benefits, but was rather intended to operate at much broader scales, by protecting large intact habitat areas. When evaluated at the statewide scale at which SGEO is intended to function, we observed that habitat and protections designated under the Wyoming SGEO were benefiting sage-grouse conservation, however the specific way in which it benefitted local populations likely varied both spatially and temporally across Wyoming. Regardless, we found that not only did the Core Area strategy include the majority of leks across the landscape, but also the majority of the male sage-grouse population as well as more birds/lek, and had a lower probability of lek collapse when compared to non-Core Area leks.
